# Alcohol Consumption and Dietary Practices in the U.S. Population: 1987 and 1992

**Published:** 1996

**Authors:** Diane K. Deitz, Gerald D. Williams, Mary C. Dufour

**Affiliations:** Diane K. Deitz, Ph.D., and Gerald D. Williams, D.Ed., are research analysts with the Alcohol Epidemiologic Data System of the National Institute on Alcohol Abuse and Alcoholism (NIAAA), which is operated by CSR, Incorporated, Washington, D.C. Mary C. Dufour, M.D., M.P.H., is deputy director of NIAAA, Bethesda, Maryland

**Keywords:** AOD consumption, nutrition, diet, health related behavior, state of health, survey, AOD use pattern, comparative study, fats, carbohydrates, vitamins, salts, mineral nutrients

## Abstract

Research indicates that the link between alcohol and poor nutrition is highly complex. Alcohol consumption is known to disrupt nutritional status via several mechanisms. One mechanism by which alcohol may affect nutrition is by inducing changes in dietary practices. Findings are presented based on 2 years of data from a national survey of health-related activities. The relationship between alcohol consumption and dietary practices—such as types of food consumed, whether vitamins were taken, belief that diet influences health, and frequency of eating out—is examined. The results show that associations exist between alcohol and diet after controlling for demographic and health variables. These findings have important implications for understanding the interaction between alcohol intake and nutritional status and the effect of this interaction on overall health.

This Epidemiologic Bulletin examines alcohol consumption and dietary practices in the general U.S. population for the years 1987 and 1992. Previous research has indicated that many complex links exist between alcohol and nutrition that affect overall physiologic functioning and health. Alcohol consumption may lead to the disruption of nutritional status via several mechanisms. One mechanism by which alcohol can affect nutritional status is in the body’s absorption, metabolism, and utilization of vitamins, proteins, fats, and carbohydrates. Another mechanism by which alcohol may affect nutritional status is in behavioral changes induced when alcohol consumption affects a person’s ability to afford both alcohol and food or when alcohol replaces the preference or need for other important foods in the diet. The purpose of the current research is to investigate the latter mechanism by examining selected dietary behaviors and beliefs associated with varying levels of alcohol consumption in a national sample of men and women.

Data for this analysis were extracted from 2 study years of cancer risk factors from the Epidemiology Study (ES) section of the National Health Interview Survey (NHIS). The study years chosen were 1987 and 1992. The NHIS is a personal interview household survey conducted on a nationwide sample of the civilian, noninstitutionalized population of the United States by the National Center for Health Statistics (NCHS). Since 1957 NCHS has conducted the survey annually. It includes a “core” set of questions on health-related variables that generally change little from year to year and supplements that can vary annually. The ES contains questions on the consumption frequency of a wide variety of different foods and beverages, including beer, wine, and liquor. Also included in the ES are questions on vitamin and mineral intake and food knowledge. The ES sample consists of adults age 18 and older randomly selected from the NHIS sample. One adult per family is selected to answer questions on the ES portion of the NHIS. The sample size of the 1987 ES was 22,080 household respondents and the sample size of the 1992 ES was 12,005 household respondents (NCHS 1988, 1994).

## Background and Understanding

A previous analysis of diet and alcohol consumption was conducted on the 1987 ES of the NHIS for an Epidemiologic Bulletin published in *Alcohol Health & Research World* in 1989. The results of these data indicated that heavier drinkers (i.e., those who reported that they drank 14 or more drinks per week) were more likely than other groups of drinkers and nondrinkers to report beliefs and behaviors that may place them at greater health-related risk resulting from poor diet (Brooks et al. 1989). Heavier drinkers were more likely to report a diet high in fat and low in fiber and to believe that diet has little effect on health. The authors concluded that these results were preliminary and that further analysis of the data was necessary before definitive conclusions could be made. The current work is intended to supplement the 1989 analysis by expanding the definition of dietary practices to include food categories that have since been found to vary in a person’s diet with level of alcohol consumption, by adding an additional study year to the analyses, and by conducting multivariate analyses that control for demographic characteristics (e.g., age and education level), body mass index (BMI) (for a definition of this and other terms, see glossary, p. 130), and current smoking status.

### Relationship of Diet and Alcohol Consumption

Many studies on diet and alcohol consumption have concentrated on the nutritional status of the alcoholic and the biomechanisms that may influence drinking behavior. In a review article on dietary interventions to reduce alcohol consumption, Litten and Allen (1992) suggested that various nutrients (e.g., carbohydrates) may modify drinking behavior by altering levels of neurotransmitters in the brain. Another line of dietary investigation has focused on the average calories consumed from carbohydrates, proteins, and fats versus the average calories consumed from alcohol. Within this subgroup of studies, a few researchers have investigated whether certain dietary practices are associated with particular levels of alcohol consumption. For example, the most consistent of the findings reported in these studies is that ingestion of sugars in the form of complex and simple carbohydrates decreases with increased alcohol intake (Colditz et al. 1991; Thomson et al. 1988; Tremblay et al. 1995), whereas ingestion of total fat increases with greater alcohol intake (Veenstra et al. 1993; Tremblay et al. 1995). Additionally, studies have posited that lifestyle factors and differences in eating patterns—such as weekend versus weekday eating, the prevalence of skipping meals, and the frequency of eating out—may differ between drinking groups (Hillers and Massey 1985; Mattes 1996; Veenstra et al. 1993). Overall, results of these studies support the notion that an increase in alcohol intake does not induce a compensatory reduction in fat (i.e., lipid) and nonalcohol energy intake but that alcohol may suppress the appetite for carbohydrates or, conversely, that carbohydrates may suppress the appetite for alcohol. Research on lifestyle factors have been scant and generally are inconclusive.

The purpose of the work reviewed was threefold: (1) to revisit the research initially conducted on these data in 1989 by examining the prevalence of certain dietary habits in relation to drinking status among demographic subgroups of the population; (2) to include measures of consumption of sweet foods, sugared drinks, and high fat foods in our analyses to add to studies citing a relationship between these foods and alcohol consumption; and (3) to conduct a multivariate analysis to eliminate the effects of variables such as education on diet and alcohol consumption.

## Methods

### Alcohol Consumption Classifications

Alcohol consumption data were derived from three items on the food frequency section of the ES. Questions were asked separately for each of the three major alcoholic beverage types: How often did you drink (beer/wine/ liquor)? On the days that you drank (beer/wine/liquor), how many (cans/bottles/glasses/drinks) did you have? Were the drinks small, medium, or large? Examples of medium drinks were provided on the survey as a reference to respondents and were defined as 12 ounces of beer, 1 medium glass of wine, or 1 shot of liquor.

Based on responses to these questions, average daily alcohol consumption was calculated for each of the alcoholic beverages. For those drinkers who did not report the drink size, a medium size was assumed. Drink size and percent of alcohol content values were consistent with the definitions used in the earlier Epidemiologic Bulletin on this subject (Brooks et al. 1989) and were based on both knowledge of current bottled beverage sizes and the percentage of alcohol used in previous analyses of NHIS surveys (Williams et al. 1994*a*). For beer, a large drink constituted 16 ounces; a medium drink, 12 ounces; and a small drink, 7 ounces. The alcohol content value used for beer was 4 percent. For wine, a large drink constituted 5 ounces; a medium drink, 4 ounces; and a small drink, 3 ounces. The alcohol content value used for wine was 15 percent. For liquor, a large drink constituted 1.5 ounces; a medium drink, 1.0 ounce; and a small drink, .75 ounce. The alcohol content value used for liquor was 45 percent.

Average daily alcohol content for each of these beverages was summed to obtain an estimate for the average daily consumption of all beverage alcohol. Based on this value, respondents were assigned to one of four drinking levels. In this analysis, the drinking classification “abstainer” refers to respondents who drank less than .01 ounce of alcohol (less than 4 drinks per year on average). “Light” refers to respondents who drank .01 to .21 ounce of alcohol (up to 3 drinks per week). “Moderate” refers to respondents who drank .22 to .99 ounce of alcohol (from 4 to 13 drinks per week). “Heavier” refers to respondents who drank 1.0 ounce or more (14 or more drinks per week or 2 or more drinks per day). The previous report excluded 201 persons classified as abstainers who reported that they had decreased their alcohol consumption.^1^ Although this manipulation refines the abstaining category by excluding former drinkers, this question was not asked in 1992 and prevented us from making similar exclusions to the data analysis.

### Dietary Classifications

A total of seven dietary behaviors were addressed in these analyses. Questions were taken from three sections of the ES including the food frequency section, the vitamin and mineral intake section, and the food knowledge section. Variations in food intake among respondents were examined for four groupings of foods from the food frequency section: fatty and fried foods, sweet foods, sugared soft drinks and beverages, and salty snacks. Foods were analyzed according to broad macronutrient (e.g., fat or carbohydrate) content versus biochemical or micronutrient content. These categories were chosen as a means of investigating food preferences according to sweet, salty, and fatty food groups. Use of these groups was intended to increase understanding of taste preferences between different drinking and nondrinking populations. Many foods contained in the sweet category, such as doughnuts, chocolate, and pastries, are also high in fat but were studied solely from the perspective of calories from carbohydrates. In support of this assessment method, Drewnowski and Schwartz (1990), by studying the sensory assessment of sugar-fat mixtures, found that study subjects judged sweeter stimuli to be lower in fat content and that the sensory assessment of fats in foods such as cakes and pastries is masked by the carbohydrate-rich taste of the food. Additional dietary variables that were examined were the prevalence of taking any vitamin/mineral supplements within the past 12 months, the belief that diet has an effect on disease and health, and the prevalence of eating out in fast food establishments or restaurants.

In the food frequency section of the survey, respondents were asked about the quantity and frequency of their typical past-year consumption of a wide variety of different foods. A listing of basic sources of fats and macronutrients in the American diet was reviewed to determine some of the major contributors of fats and carbohydrate intake as measured in general population studies (Block et al. 1985). This article quantifies nutrient sources using dietary data from the second National Health and Nutrition Examination Survey to provide information to epidemiologists investigating dietary etiologies. From these data, lists of foods that typified broad dietary classifications, such as highly fatty foods and sweet or sugary foods, were constructed. Production of exhaustive listings of items within each food category was beyond the scope of this article and was not attempted, nor was it possible to control for food products that might have been low in fat, such as low-fat cheese. Finally, foods contained in the salty snacks category, such as chips and nuts, are high in fat but were studied from the perspective of sodium intake.

The fatty and fried foods measure was obtained by summing the total of several foods, including hamburgers, beef, hot-dogs, lunch meats, whole milk, fried fish, fried chicken, bacon, sausage, eggs, butter, margarine, cheese or cheese spreads, and peanuts or peanut butter. Construction of the sweet foods and sugars measure was conducted in the same manner and included ice cream, pie, doughnuts, cookies, cake or pastry, and chocolate candy. The sugared drinks measure was defined by drinking nondiet soda or sugared soft drinks and by adding sugar to coffee or tea. The salty snacks measure was derived from one question asking how often salty snacks were consumed. Although not specifically listed with the question, this category attempted to measure foods such as potato chips, pretzels, and salted nuts. Each item was coded for the total number of times it was consumed per year based on responses to the number of servings reported during specified time periods. The sum of these food categories was divided by 52 to obtain an average number of servings consumed per week.

From responses to these questions, each dietary class was divided into two mutually exclusive categories. First, approximate cut-points for the dietary variables (i.e., the consumption level that divided one category from the other) were determined by examining the distributions of the data and finding groupings for which at least 20 percent of the population fell into each outcome category. The categories were then further refined based on ease of interpretation (i.e., 7 servings per week translates into an average of 1 serving per day). The final classifications were divided as follows:

- The fatty and fried foods measure was dichotomized into respondents consuming 21 or more servings of these foods per week or those consuming less than 21 servings per week.
- The sweet foods and the sugared drinks measures were dichotomized into respondents consuming 7 or more of these foods per week or those consuming less than 7 servings of these foods.
- The salty snacks measure was dichotomized into respondents who consumed these foods 1 or more times per week and those not consuming these foods weekly.
- Eating at restaurants or fast food establishments was dichotomized into respondents eating out 2 or more times per week and those eating out less than 2 times per week.
- The dietary belief question was dichotomized into respondents who believed that diet has little effect on disease and those who believed that diet can reduce disease.
- Finally, the vitamin and mineral supplement was dichotomized into respondents who reported that they had taken vitamins and or minerals within the past 12 months and those who had not taken any vitamin or mineral supplements in the past 12 months.

### Data Analysis

As a first step in understanding the relationship of alcohol consumption to diet, descriptive analyses of the data were calculated. Basic characteristics of the study sample are provided as unweighted numbers and weighted percentages. Weights are applied to the data to adjust for sampling procedures used in the collection of NHIS data. Results are based on a sample size of 22,080 respondents in 1987 and 12,005 respondents in 1992. Percentages are used in all comparison analyses and are generated from weighted sample data. The NHIS oversamples racial/ethnic minorities and older persons to ensure the reliability of estimates for these subgroups. It was necessary, therefore, to adjust for these data collection methods to obtain estimates representative of the general population. Appropriate weights for such adjustments were provided with the NHIS and were used in this research.

After observing the relationships between alcohol consumption levels and the dichotomized dietary categories (i.e., the descriptive data in tables 2A and 2B), logistic regression analyses were conducted to test for the potential significant effect of alcohol on each of the selected dietary practices. Two processes were used in conducting the logistic analyses. First, alcohol was tested alone for its effect on each of the dietary outcomes. The results are presented as the unadjusted odds ratios and are termed “unadjusted” because they do not take into account any other variables that may have an effect on alcohol and diet. Second, multiple logistic regression analyses were conducted that accounted for the variables that may influence the relationship between alcohol and diet (i.e., the confounding variables). This technique is termed “adjusted,” because it allows for the determination of alcohol consumption’s effect on diet independent of confounding variables.

The confounding variables controlled for were age, sex, education, BMI, and smoking status. Age, education, and alcohol consumption were entered as categorical variables with dummy variables; and sex, BMI, and smoking status were entered as dichotomous variables. The group on which the odds ratios were compared (i.e., the reference grouping) for the three age levels was “the 18 to 29 year olds”; for the four groupings of education, the reference group was “less than a high school education”; for the four levels of alcohol consumption, the reference group was “abstainers”; for sex, the reference group was “females”; for BMI, the reference group was “not overweight”; and for smoking, the reference group was “not currently smoking.” BMI was derived by using estimation procedures developed by the National Health and Nutrition Examination Survey II (NHANESII). The cut-points for overweight were 27.8 or greater for men and 27.3 or greater for women (these values approximate the sex-specific 85th percentile of BMI estimated from the NHANESII). The statistical package SUDAAN was used in all multivariate analyses because of its ability to calculate variance estimations from complex sample designs such as that used by the NHIS (Shah et al. 1992).

### Limitations

One limitation of this study was the method used to collect dietary information. Dietary intake of foods is based on the respondents’ recall for a time period of up to 1 year. Seasonal variations in food intake and variations induced by various life changes could not be accounted for in this analysis. Additionally, there may be many “hidden” calories not detected by the food histories. For example, a person who ate fettuccine Alfredo every week may have listed this food selection as a serving of pasta, omitting the high intake of fat and additional calories contained in the sauce. Consequently these data lack the precision of an experimental or more controlled dietary intake study. These data do, however, give a good estimation of “typical” food and alcohol intake.

Another limitation of the study was that the analyses were restricted to questions contained in both the 1987 and 1992 surveys. The original analyses of these data in 1989 contained several dietary variables that were not included in the current research. For example, the 1989 study analyzed whether respondents ate fewer than two meals per day on weekdays, whether they perceived their diet to be low in fiber or high in fat, and whether they reported lasting and major changes in diet for health reasons. Unfortunately, the 1992 survey did not repeat these questions so they could not be included in the current analysis. It also should be noted that data from each of the 2 study years are cross-sectional, thereby precluding longitudinal analysis of the data.

## Results

### Drinking Pattern Changes, 1987–1992

Table 1 presents the unweighted frequencies and weighted percentages of drinking classifications by sex, age, and education. Consistent with findings from other national data on alcohol consumption, the prevalence of abstainers increased with age, and the prevalence of light, moderate, and heavier drinkers decreased with age. Females were more likely than males to be abstainers or light drinkers, and males were more likely than females to be heavier drinkers. The prevalence of abstainers increased as education decreased, and the prevalence of light and moderate drinking increased with higher educational achievement.

Comparison of data on drinking status from 1987 to 1992 indicates that the overall prevalence of abstention from alcohol increased from 41 percent in 1987 to 47 percent in 1992. Accordingly, all categories of current drinking were lower in 1992 compared with 1987. The decrease in current drinking status was similar across age, sex, and education categories. This finding was consistent with other analyses conducted by the National Institute on Alcohol Abuse and Alcoholism’s (NIAAA’s) Alcohol Epidemiologic Data System (AEDS) and reflects a general decline in alcohol consumption since the 1980’s (Williams et al. 1994*b*).

### Diet and Alcohol Consumption, 1987–1992

Tables 2A and 2B present the prevalence of selected dietary habits separated by sex, age, and education for the survey years of 1987 and 1992. Descriptive data for the dietary outcomes by drinking status are briefly discussed to give an overview of the relationship of diet to alcohol consumption prior to controlling for all of the selected demographic and health variables. Descriptive data are not described in detail because many of the outcome variables are influenced by factors that require multivariate analysis for appropriate interpretation.

Table 2A presents the prevalence of eating 21 or more servings of fat per week, eating 7 or more servings of sweet foods per week, drinking 7 or more sweetened beverages per week, and eating salty snacks weekly by levels of drinking status and by population totals. The prevalence of eating 21 or more servings of fat per week decreased from 1987 to 1992 and was consistent across drinking categories. The prevalence of eating more fatty foods tended to be less in the light drinkers compared with the abstainers, higher in the moderate drinkers, and highest in the heavier drinkers. The prevalence of eating 7 or more servings of sweet foods per week generally decreased as consumption of alcohol increased. The biggest differences in eating sweet foods were between the abstainers and light drinkers and between the moderate and heavier drinkers. The prevalence of drinking sweetened beverages was fairly consistent across drinking categories. The biggest differences in prevalence of this behavior were observed across demographic strata, with the prevalence of drinking sweetened beverages decreasing with female sex and with higher education. Finally, the prevalence of eating salty snacks generally increased as the levels of alcohol consumption increased. Overall, eating salty snacks was lower in the abstainers and light drinkers compared with the moderate and heavier drinkers.

Table 2B presents data on the prevalence of eating out at least twice per week, taking any vitamin or mineral supplements, and the belief that diet has little effect on disease, grouped according to levels of drinking status and population totals. Prevalence of eating out consistently increased as levels of alcohol consumption increased. The only exception to this pattern was in the 60+ year-old strata, where the highest prevalence of eating out was in the light drinkers. Eating out prevalence varied considerably by demographic characteristics, with the highest prevalence among men, younger individuals, and those with higher education.

The prevalence of not taking vitamin or mineral supplements was lowest among the light and moderate drinkers and, conversely, highest among the abstainers and heavier drinkers. A minority of the population believed that diet did not have an effect on disease (8 percent in 1987 and 10.4 percent in 1992). The prevalence of believing that diet had little effect on disease generally was highest in the heavier drinkers. Comparing the prevalence of believing that diet has little effect on disease in the total population to the heavier drinkers showed a consistently higher prevalence for heavier drinkers across almost all demographic strata.

Table 3 and the figure present the odds ratios and the 95-percent confidence intervals for levels of alcohol consumption and dietary practices. The unadjusted odds ratios in table 3 are data estimates calculated prior to controlling for age, sex, education, BMI, and smoking status. The adjusted odds ratios are data estimates calculated with all of the control variables entered into the model. The adjusted odds ratios presented in table 3 and the figure give a good estimation of the independent effects of alcohol consumption on the various dietary behaviors. The odds ratios for each level of alcohol consumption are given in relation to abstention, which is the reference category.

### Sweet Foods and Alcohol

In both 1987 and 1992, the odds of consuming greater amounts of sweet foods decreased as alcohol consumption increased. In 1987 light drinkers were not significantly different from abstainers in their consumption of sweet foods; however, in 1992 light drinkers were less likely to consume these foods. In both study years, moderate and heavier drinkers were significantly less likely to consume sweet foods when compared with abstainers. Heavier drinkers were 27 percent less likely to consume sweets in 1987 and 32 percent less likely in 1992. This finding is consistent with previous research on consumption of sweet foods and alcohol and supports an inverse relationship between the two dietary behaviors.

### Fatty Foods and Alcohol

Alcohol consumption was weakly related to eating fatty foods in this data analysis. In 1987 heavier drinkers were significantly more likely to consume more fatty foods compared with abstainers, and in 1992 light drinkers were less likely to consume fatty foods. In 1992 no significant differences were observed for the heavier drinkers when compared with abstainers. The tendency for heavier drinkers to eat more fatty foods was present in both study years, but the relationship was not a strong one. Overall, these data are not strongly supportive of research documenting a positive relationship between consumption of alcohol and consumption of total fats (mentioned earlier).

### Sweetened Drinks and Alcohol

The consumption of sweetened soft drinks and beverages decreased as consumption of alcohol increased. All levels of alcohol consumption were associated with significantly lower odds of consuming sweetened drinks compared with abstainers for both 1987 and 1992. Heavier drinkers were 22 percent less likely to consume sweetened drinks in 1987 and 20 percent less likely in 1992. This finding was consistent with the finding that consumption of sweet foods decreased as alcohol consumption increased and, therefore, strengthened the credibility of this association. As shown in the descriptive data (table 2), and in comparing the unadjusted odds ratios to the adjusted odds ratios, consumption of sweetened drinks appeared to be strongly influenced by demographic variables.

**Figure:**
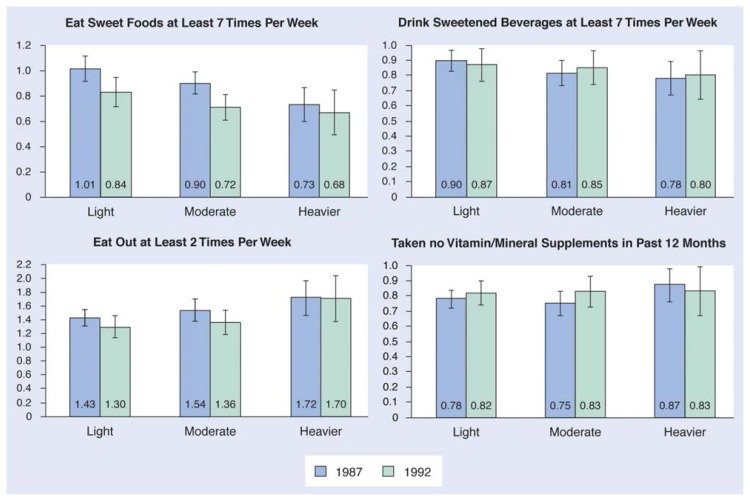
Odds ratios of selected dietary practices by alcohol consumption level with 95-percent confidence intervals (CI). Data for this figure are also provided in table 3. Vertical lines indicate the 95-percent CI.

### Salty Snack Foods and Alcohol

Consumption of salty snacks generally increased with consumption of alcohol. A significantly greater consumption of salty snacks was observed among moderate drinkers compared with abstainers for both 1987 and 1992. Moderate drinkers were 27 percent more likely to eat salty snacks in 1987 and 39 percent more likely in 1992. A strong relationship between this variable and alcohol consumption was observed prior to adjustment of all of the control variables. Investigation of the full model indicated that consumption of salty snacks decreased considerably as age increased. Although an exploration of the reasons behind this finding are beyond the scope of this article, potential explanations for the reduction in consumption of salty snacks may be as follows: for health reasons (e.g., high blood pressure), a reduction in going to parties and bars where these foods are frequently served, or changes in food preferences that may occur with age.

### Eating Out and Alcohol

Eating out increased consistently as levels of alcohol consumption increased. Results were significant at all levels of consumption for both study years. Overall, heavier drinkers were nearly twice as likely to eat out (72 percent higher in 1987 and 70 percent higher in 1990) when compared with abstainers. As seen with the descriptive data, age, sex, and education all were associated with eating out; however, control for these variables did not diminish the relationship of alcohol to eating out. Although speculative, potential reasons for this finding include lifestyle and social influences, which are discussed in greater detail in the conclusions section.

### Vitamin/Mineral Supplements and Alcohol

Taking vitamin and mineral supplements was significantly more likely for all levels of current drinkers compared with abstainers for both study years. Although little difference among levels of drinking was observed in 1992, moderate and light drinkers were slightly more likely to take vitamin or mineral supplements compared with heavier drinkers in 1987. Overall, these data indicate that individuals who currently drank alcohol were more likely to supplement their diets with vitamins and/or minerals than were abstainers.

### Belief That Diet Has Little Effect on Health and Alcohol

The relationship of believing that diet has little effect on disease and consumption of alcohol was inconsistent in this data analysis. In 1987 heavier drinkers were significantly more likely to have this belief; however, in 1992 the opposite finding was observed. That is, heavier drinkers were more likely to believe that diet did affect disease. Light and moderate drinkers also tended to believe that diet affected disease; however, the relationship was not a strong one.

## Conclusions

This study examined the relationship between alcohol consumption and dietary practices for 2 study years of a nationally representative survey of the U.S. population. Alcohol was found to be independently associated with several dietary practices after controlling for important demographic and health variables. The results of these data allow us to draw several conclusions on the contribution of alcohol to diet.

First, these data demonstrate the expected inverse relationship between alcohol intake and consumption of sweet foods and sugared beverages. The concordance of results between the 2 study years and the two outcome variables (i.e., sweet foods and sweetened beverages) provide considerable support for the validity of this finding. In addition, the inverse relationship found in this study agrees with the relationship that has generally been reported in the literature (e.g., Colditz et al. 1991; Thomson et al. 1988; Tremblay et al. 1995). The precise mechanism by which alcohol may satiate the craving or need for carbohydrates has yet to be determined in experimental studies. Previous research has indicated that recovering alcoholics or alcoholics who have stopped drinking appear to develop an appetite for cake, ice cream, chocolate, and candy (Colditz et al. 1991). Additionally, a study on alcoholic patients treated in an outpatient clinic found that patients were more likely to remain sober when they reported diets containing twice as much sugar added to beverages and greater intake of carbohydrates (Yung et al. 1983). A potential explanation for these findings is in the literature on food cravings, where it has been hypothesized that dysphoric moods (e.g., abnormal feelings of discontent or anxiety) elicit carbohydrate craving and that carbohydrate intake, in turn, can ameliorate psychological symptoms such as depression (Wurtman and Wurtman 1986). Thus, it is possible that recovering alcoholics stave off the temporary depression that can occur with abstinence by eating more carbohydrates in the form of sweets. Results of this study also indicated that consumption of sweetened drinks is strongly influenced by demographic variables and requires control of these variables in population-based studies.

Second, exploration of the relationship between alcohol consumption and consumption of fatty foods indicated that moderate and heavier drinkers tended to eat more fatty foods; however, the relationship was not a strong one. A stronger relationship was observed in the bivariate and trend analyses, which indicated that overall consumption of fat decreased substantially from 1987 to 1992. These data reflect the recent national trend in consuming less fats and may overshadow a relationship between alcohol and consumption of fatty foods. Additionally, total consumption of fat may not have been adequately measured in this analysis. The study did not attempt to produce an exhaustive listing of fatty foods in its measure; rather, several foods that obviously are high in fat calories were chosen. In addition, fat is hidden in many food choices, such as dressings, gravies, and sauces and therefore could not be measured by these data. Despite the potential shortcomings of our approach, other studies have found fat intake to be unchanged (Orozco et al. 1991) or decreased (Hillers and Massey 1985; Jones et al. 1982) in relation to alcohol consumption. Further exploration of this relationship is merited, because both fat intake and alcohol consumption are important predictors of cardiovascular health. Moderate consumption of alcohol has been related to a lower risk of coronary artery disease in numerous studies (Marmot 1981; Veenstra et al. 1991; Klatsky 1994). Two hypotheses that are used in explaining this finding are as follows: (1) moderate alcohol consumption is associated with a healthier lifestyle, and (2) alcohol exerts a direct beneficial effect on physiologic mechanisms (e.g., cholesterol metabolism or platelet stickiness). Results of our study—which indicate that moderate drinkers do not eat fewer fatty foods than abstainers—do not appear to support the first hypothesis of a healthier lifestyle as demonstrated by a decrease in other risk factors, such as a lower intake of fatty foods.

Consumption of alcohol was associated with increased consumption of salty snacks and with the incidence of eating out for both study years. Although this analysis did not investigate these associations in more detail, these findings are intriguing and may merit further investigation. Both of these dietary behaviors may be strongly influenced by lifestyle and social variables. The influence of social situations on the frequency of drinking alcohol and on the health consequences resulting from this behavior was recently explored during an international conference on the social and health effects of different drinking patterns. Trevisan and colleagues (1995) found that drinking alcohol with meals resulted in smaller relative risks for mortality than drinking at other times. It may be that both the amount of alcohol consumed and the health effects of alcohol differ by social situation (i.e. going out to a restaurant with the primary motive to eat versus going out to a bar with the primary motive to drink alcohol). The association between alcohol consumption and eating meals in a restaurant or eating salty snacks may be better understood once variables, such as the frequency of going to parties, going out to bars, and engaging in various social situations, are taken into account.

The data on drinking and taking vitamin or mineral supplements generally support a positive relationship between these two behaviors. In general, all levels of current drinkers were more likely to take vitamins compared with abstainers. This finding may be indicative of a positive health behavior among drinkers. However, less encouraging is the finding that heavier drinkers do not take supplements with a greater frequency than moderate and light drinkers. Although vitamin and mineral supplements are not needed for persons whose nutritional needs are met through adequate dietary habits, heavier drinkers have a greater risk of malnutrition (Feinman 1989) and may, therefore, require supplementation more frequently than abstainers and nonheavy drinkers.

Finally, data on the relationship between drinking and the belief that diet has an effect on health were inconsistent. This outcome was included in the 1989 *Alcohol Health & Research World* article using the 1987 NHIS database and was included as a followup to this analysis. In 1987 heavier drinkers were significantly more likely to hold the belief that diet did not affect health, but in 1992 the opposite finding was observed. There are several potential explanations for this inconsistency. First, only a minority of individuals believed that diet did not affect health (8 percent in 1987 and 10 percent in 1992). Additionally, it is unclear whether people consider alcohol consumption to be subsumed under the general category of diet and whether the question from the survey refers to a positive or a negative effect of diet on health.

In summary, results of this study add to an understanding of alcohol and dietary behaviors and contribute to a growing literature addressing this issue. Associations were found between alcohol and diet even after controlling for demographic and health variables known to co-vary with diet. Further research is needed to better understand the complex interaction of alcohol intake on nutritional status and the effect of this interplay on health as well as to determine the myriad social influences that impact alcohol and diet.

## Figures and Tables

**Table 1:** Weighted Percentages and Unweighted Sample Sizes of Alcohol Consumption Levels by Sex, Age, and Education, 1987 and 1992 NHIS^1^

	Alcohol Consumption Levels^2^							

	Abstainer		Light		Moderate		Heavier	
	1987	1992	1987	1992	1987	1992	1987	1992
**Total**								
Weighted percentage	41.2	47.3	29.6	26.2	21.0	19.5	8.2	7.0
Unweighted sample size	(9,230)	(5,683)	(6,352)	(3,029)	(4,458)	(2,255)	(1,786)	(795)

**Men**								
All men	30.8 (2,747)	36.5 (1,776)	27.4 (2,427)	25.7 (1,270)	27.8 (2,522)	26.0 (1,353)	14.0 (1,349)	11.8 (616)
18–39 years	23.8 (993)	29.9 (690)	29.3 (1,285)	27.1 (672)	31.2 (1,432)	29.6 (761)	15.8 (772)	13.3 (349)
40–59 years	31.5 (780)	37.2 (536)	26.7 (673)	26.1 (377)	27.3 (688)	24.9 (390)	14.4 (386)	11.8 (182)
60+ years	47.2 (974)	51.8 (550)	23.7 (469)	21.4 (221)	20.2 (402)	18.8 (202)	9.0 (191)	8.0 (85)

**Women**								
All women	50.6 (6,483)	57.2 (3,907)	31.6 (3,925)	26.6 (1,759)	14.8 (1,936)	13.6 (902)	3.1 (437)	2.7 (179)
18–39 years	40.5 (2,387)	48.1 (1,483)	38.2 (2,310)	32.3 (1,000)	17.8 (1,156)	16.5 (512)	3.5 (255)	3.2 (101)
40–59 years	52.6 (1,592)	57.6 (1,019)	30.4 (962)	26.3 (468)	13.7 (446)	13.7 (243)	3.3 (117)	2.5 (50)
60+ years	68.8 (2,504)	73.9 (1,405)	19.5 (653)	16.3 (291)	10.0 (334)	8.0 (147)	1.8 (65)	1.9 (28)

**Education**								
< High school graduate	57.5 (3,149)	66.7 (1,788)	21.4 (1,087)	15.6 (414)	13.9 (694)	12.3 (314)	7.2 (357)	5.5 (140)
High school graduate	42.3 (3,575)	49.8 (2,151)	30.1 (2,441)	25.3 (1,063)	19.3 (1,539)	17.5 (716)	8.3 (660)	7.4 (307)
Some college	32.4 (1,409)	38.1 (978)	33.7 (1,446)	30.8 (747)	25.0 (1,039)	22.8 (564)	9.0 (426)	8.3 (195)
College graduate	27.9 (1,097)	32.6 (766)	34.5 (1,378)	33.8 (805)	29.2 (1,186)	27.0 (661)	8.5 (343)	6.6 (153)

1 NHIS = National Health Interview Survey.

2 Alcohol consumption levels are defined as follows: abstainer = < 0.01 ounce of alcohol (< 4 drinks per year); light = 0.01–0.21 ounce of alcohol (up to 3 drinks per week); moderate = 0.22–0.99 ounce of alcohol (4–13 drinks per week); and heavier = 1.0 ounce or more of alcohol (14 or more drinks per week or 2 or more drinks per day).

**Table 2A:** Prevalence^1^ of Selected Dietary Habits by Sex, Age, Education, and Alcohol Consumption Level, 1987 and 1992 NHIS^2^

	Alcohol Consumption Level^3^									

	Abstainer (%)		Light (%)		Moderate (%)		Heavier (%)		Total (%)	
	1987	1992	1987	1992	1987	1992	1987	1992	1987	1992
**Report Diet High in Fat (eat 21 or more servings of fatty foods per week)**										
Total	51.4	41.1	51.6	38.8	56.6	44.4	65.5	52.9	53.9	42.6
Male	59.6	49.6	59.4	46.6	62.4	48.3	68.6	55.7	61.6	49.2
Female	47.0	36.1	45.5	32.1	46.9	37.7	52.6	41.5	46.7	35.4
18–39 years	59.3	50.6	56.2	42.7	62.0	50.9	73.2	61.2	60.2	49.2
40–59 years	47.5	37.3	46.3	33.6	49.6	37.6	56.0	45.4	48.3	37.0
60+ years	45.4	32.6	44.1	35.8	48.8	33.4	52.9	36.9	46.0	33.5
< High school graduate	54.2	46.3	63.2	46.6	68.7	55.1	73.8	53.9	59.5	47.9
High school graduate	53.1	43.3	54.1	46.7	62.9	51.7	72.0	62.8	56.8	47.1
Some college	49.8	36.1	49.2	37.1	54.6	44.9	63.5	55.4	52.0	40.0
College graduate	41.1	30.1	40.3	26.1	42.4	30.5	45.1	28.6	41.5	28.7

**Report Diet High in Sweet Foods (eat 7 or more servings of sweet foods per week)**										
Total	27.1	25.2	27.5	25.9	22.5	20.7	23.5	21.0	26.4	23.3
Male	33.3	28.3	31.1	27.8	26.1	21.9	24.3	21.4	29.6	25.2
Female	23.8	23.4	24.7	22.9	19.4	18.6	19.9	19.3	23.6	21.6
18–39 years	27.4	27.7	27.7	24.6	22.7	21.8	24.4	20.2	26.4	24.2
40–59 years	25.0	22.3	25.4	22.8	20.0	16.9	18.1	20.1	23.9	20.5
60+ years	28.7	24.9	30.0	36.3	26.7	24.4	31.1	25.9	29.7	25.2
< High school graduate	27.3	25.8	29.5	30.8	22.3	24.3	30.1	22.6	28.0	24.9
High school graduate	28.6	26.1	28.1	27.2	25.2	23.0	24.0	24.3	27.6	25.2
Some college	25.0	23.2	27.8	25.0	20.2	19.3	20.5	18.4	25.3	21.0
College graduate	24.8	23.7	24.5	22.0	21.1	17.6	18.6	16.0	23.2	20.7

**Report Diet High in Sweetened Drinks (drink 7 or more sweetened beverages per week)**										
Total	48.9	48.1	49.6	50.1	46.7	48.8	54.5	52.7	50.3	49.1
Male	56.4	52.6	57.4	56.0	52.1	53.6	58.0	56.6	57.2	54.0
Female	44.9	45.5	43.6	40.1	41.9	40.5	40.3	36.9	44.1	44.6
18–39 years	60.0	58.2	55.8	55.8	53.9	54.4	61.3	60.4	58.0	56.8
40–59 years	48.1	46.3	44.8	45.2	41.6	46.2	48.3	48.4	47.0	46.1
60+ years	36.3	36.9	35.9	36.9	31.8	32.7	38.9	32.2	37.5	36.8
< High school graduate	52.1	50.0	60.7	63.7	53.9	63.8	67.1	65.2	57.3	54.1
High school graduate	50.9	51.4	53.4	55.1	53.6	56.9	60.2	59.8	53.6	54.4
Some college	45.2	42.3	47.6	47.6	46.0	44.9	49.6	45.5	47.5	44.9
College graduate	39.2	42.0	36.3	37.0	34.6	35.9	34.9	37.0	37.7	38.4

**Report Eating Salty Snacks (eat salty snacks at least weekly)**										
Total	43.4	48.5	52.1	56.4	57.6	62.8	59.0	59.2	49.5	54.4
Male	46.0	50.8	53.3	56.9	58.4	64.5	60.0	61.0	52.5	57.5
Female	41.9	47.1	51.2	55.7	56.8	59.9	55.1	52.1	46.9	51.5
18–39 years	61.5	63.5	61.6	65.3	65.6	71.3	70.4	68.9	62.9	66.3
40–59 years	43.8	49.9	47.6	52.1	55.8	57.1	47.2	54.8	46.5	53.1
60+ years	21.1	27.4	26.3	30.9	33.1	41.7	35.8	30.9	23.9	30.4
< High school graduate	34.2	36.8	44.7	46.1	46.2	59.9	52.6	49.4	38.9	41.8
High school graduate	48.1	54.4	54.9	59.4	61.9	68.3	61.3	61.7	53.0	59.2
Some college	49.3	55.3	55.0	59.1	56.3	61.0	63.8	65.7	54.5	57.8
College graduate	44.4	48.3	49.7	56.1	58.2	59.2	55.5	54.0	50.2	55.0

1 Prevalence is estimated by weighted percentages.

2 NHIS = National Health Interview Survey.

3 Alcohol consumption levels are defined as follows: abstainer = < 0.01 ounce of alcohol (< 4 drinks per year); light = 0.01–0.21 ounce of alcohol (up to 3 drinks per week); moderate = 0.22–0.99 ounce of alcohol (4–13 drinks per week); and heavier = 1.0 ounce or more of alcohol (14 or more drinks per week or 2 or more drinks per day).

**Table 2B:** Prevalence^1^ of Selected Dietary Habits by Sex, Age, Education, and Alcohol Consumption Level, 1987 and 1992 NHIS^2^

	Alcohol Consumption Level^3^									

	Abstainer (%)		Light (%)		Moderate (%)		Heavier (%)		Total (%)	
	1987	1992	1987	1992	1987	1992	1987	1992	1987	1992
**Report Eating Out at Least Twice Per Week**										
Total	39.0	42.8	54.9	56.7	58.8	59.7	61.8	64.4	49.3	51.3
Male	45.7	50.0	60.0	59.2	62.0	63.5	63.3	65.5	56.1	57.7
Female	35.3	38.6	50.8	54.6	53.5	53.2	55.7	59.6	43.1	45.4
18–39 years	49.0	51.1	60.7	62.2	67.2	66.5	70.6	76.6	59.0	60.0
40–59 years	41.3	45.0	52.9	58.1	53.9	56.4	57.2	57.6	48.2	51.5
60+ years	25.0	29.6	37.6	35.2	36.0	40.1	34.1	31.4	29.3	32.0
< High school graduate	25.8	27.8	37.0	34.5	41.4	44.6	45.0	52.1	31.3	32.3
High school graduate	41.5	45.6	53.5	53.5	54.9	56.0	61.4	62.8	48.9	50.7
Some college	49.2	51.0	61.4	61.0	64.7	62.3	70.1	69.2	58.5	58.1
College graduate	52.8	56.1	64.5	67.6	69.4	68.8	71.1	71.6	62.7	64.4

**Did Not Take Any Vitamin/Mineral Supplements in Past 12 Months**										
Total	52.0	55.6	44.3	49.4	45.4	51.6	54.0	56.0	48.6	53.4
Male	59.6	61.4	53.1	59.1	52.3	57.7	57.7	59.0	55.6	59.7
Female	47.9	52.2	37.4	40.9	33.7	41.1	38.6	43.9	42.2	47.7
18–39 years	50.0	56.6	43.5	49.6	44.9	52.3	55.7	56.5	47.2	53.7
40–59 years	53.3	54.5	44.5	49.1	45.4	52.6	52.4	55.0	49.2	52.9
60+ years	53.3	55.4	46.4	49.4	47.3	47.0	50.1	56.2	50.9	53.5
< High school graduate	62.2	64.0	56.1	56.2	59.3	68.2	68.5	66.2	61.0	63.6
High school graduate	50.7	55.4	46.5	55.1	48.2	56.7	57.4	59.7	49.5	56.1
Some college	44.1	48.0	37.4	44.1	40.5	47.3	45.5	52.3	41.1	47.2
College graduate	39.8	47.7	38.1	43.5	37.6	41.8	40.8	44.4	38.7	44.4

**Believe Diet Has Little Effect on Disease**										
Total	8.2	11.6	6.7	8.2	7.3	9.6	12.9	12.8	8.0	10.4
Male	9.1	14.4	7.9	10.2	7.8	10.8	13.0	12.1	9.0	12.2
Female	7.7	10.0	5.8	6.4	6.4	7.6	12.6	15.5	7.1	8.9
18–39 years	7.7	13.3	6.5	9.0	7.0	10.5	11.5	11.4	7.5	11.3
40–59 years	8.2	9.9	6.1	6.9	7.0	8.3	13.0	11.7	7.8	9.0
60+ years	8.8	11.0	8.6	7.8	8.9	9.2	18.7	21.3	9.4	10.7
< High school graduate	11.2	15.3	10.8	13.5	11.2	12.9	21.9	21.7	12.0	15.1
High school graduate	7.9	12.4	7.1	9.7	8.8	11.4	15.5	11.8	8.5	11.6
Some college	5.6	8.4	6.3	7.9	5.2	8.0	7.1	13.3	5.9	8.6
College graduate	5.6	6.7	3.5	4.2	5.1	7.7	5.8	7.6	4.8	6.3

1 Prevalence is estimated by weighted percentages.

2 NHIS = National Health Interview Survey.

3 Alcohol consumption levels are defined as follows: abstainer = < 0.01 ounce of alcohol (< 4 drinks per year); light = 0.01–0.21 ounce of alcohol (up to 3 drinks per week); moderate = 0.22–0.99 ounce of alcohol (4–13 drinks per week); and heavier = 1.0 ounce or more of alcohol (14 or more drinks per week or 2 or more drinks per day).

**Table 3:** Unadjusted and Adjusted^1^ Odds Ratios (OR’s) and 95-Percent Confidence Intervals (CI) for Levels of Alcohol Consumption^2^ and Dietary Practices,^3^ 1987 and 1992 NHIS^4^

	1987			1992		
	
	Unadjusted OR	Adjusted OR	Adjusted 95% CI	Unadjusted OR	Adjusted OR	Adjusted 95% CI
**Sweet Foods (7 or more servings per week)**						
Light	1.02	1.01	0.92–1.11	0.86	0.84	0.75–0.95
Moderate	0.94	0.90	0.81–0.99	0.78	0.72	0.63–0.82
Heavier	0.82	0.73	0.63–0.86	0.79	0.68	0.55–0.85

**Fatty Foods (21 or more servings per week)**						
Light	0.98	0.92	0.85–1.00	0.80	0.86	0.77–0.97
Moderate	1.15	1.03	0.93–1.14	1.13	0.97	0.86–1.09
Heavier	1.70	1.25	1.09–1.43	1.65	1.08	0.91–1.29

**Sugared Drinks (7 or more servings per week)**						
Light	1.03	0.90	0.83–0.97	0.94	0.87	0.77–0.98
Moderate	1.04	0.81	0.73–0.89	1.03	0.85	0.76–0.96
Heavier	1.25	0.78	0.68–0.89	1.20	0.80	0.67–0.96

**Salty Snacks (at least 1 serving per week)**						
Light	1.42	1.08	1.00–1.17	1.45	1.16	1.03–1.31
Moderate	1.70	1.27	1.15–1.40	1.80	1.39	1.24–1.56
Heavier	1.88	1.35	1.18–1.55	1.54	1.14	0.94–1.39

**Eating Out (2 or more times per week)**						
Light	1.90	1.43	1.33–1.55	1.75	1.30	1.15–1.46
Moderate	2.23	1.54	1.39–1.70	1.97	1.36	1.21–1.53
Heavier	2.53	1.72	1.50–1.97	2.41	1.70	1.42–2.03

**No Vitamin Supplements (in past 12 months)**						
Light	0.73	0.78	0.72–0.84	0.78	0.82	0.74–0.90
Moderate	0.76	0.75	0.68–0.83	0.85	0.83	0.74–0.93
Heavier	1.08	0.87	0.77–0.98	1.02	0.83	0.69–0.99

**Dietary Beliefs (believe diet has little effect on disease)**						
Light	0.81	0.90	0.77–1.06	0.68	0.71	0.59–0.87
Moderate	0.89	0.96	0.81–1.15	0.81	0.78	0.63–0.97
Heavier	1.67	1.61	1.30–2.00	1.12	0.90	0.67–1.20

1 The control variables of sex, age, education, body mass index, and smoking status were entered into the adjusted models.

2 “Abstainer” is the reference category for the “levels of alcohol consumption.”

3 The number of servings per week is based on an average of yearly consumption.

4 NHIS = National Health Interview Survey.

## Footnotes

1 Persons in the 1987 data who currently drank less than 4 drinks per year but had consumed 5 or more drinks per day at one time were classified as former drinkers and excluded in the analysis of the 1987 data (201 persons). This calculation was not possible with the 1992 data and consequently was not done for these analyses (i.e., the 201 people were included in the 1987 population).

## Glossary

Note: Some of the glossary terms have been specifically tailored as an explanation of how they are used in this article.

Body mass index (BMI): A measure used to define an individual as overweight. BMI is calculated by dividing weight in pounds by height in inches and multiplying by a sex-specific factor (cut-points for this analysis were derived from the National Health and Nutrition Examination Survey II [NHANES II] 85th percentile of BMI).
Categorical variables: Variables with more than two mutually exclusive groupings or categories.
Confidence interval: The 95-percent confidence interval gives a parameter within which one is 95-percent certain that the true estimate (which is unknown) lies. The intervals surrounding an *odds ratio* are statistically significant if the interval values are above or below the value of 1.0 (i.e., 1.0 is the value of no association).
Confounding: Refers to the possibility that the variables thought to represent a particular cause-and-effect association are related to another set of variables.
Dichotomous variables: Variables having only two mutually exclusive groupings or categories.
Dummy variables: Variables with assigned values (e.g., 1 indicates the presence of a condition and 0 indicates the absence of a condition) that have been constructed to allow a comparison of categories within a given population.
Logistic regression analysis: A regression technique used with *dichotomous outcome variables.* Regression analysis is the analysis of the linear relationship (or association) of conditions to outcomes in a given population.
Multivariate analysis: Concurrent measurement or analysis of three or more variables.
Odds ratio: The ratio of probability that a factor will be present in two populations, each of which has different characteristics or exposures to a risk factor (e.g., the incidence rate of lung cancer for populations exposed to cigarette smoking and those who were not exposed).
Variance estimation: A measure of variation allowing for the estimation of the amount of dispersion around a measure of data, such as a percentage or mean.
Weighted percentage: Percentages that have been adjusted to allow for differences in sampling techniques. Weighting ensures that the proportion of the groups being studied is as close as possible to the actual proportion in the population.
